# Relationship between sugar-sweetened beverage and ultra-processed food intake: impact on leptin/adiponectin ratio in adolescents with obesity

**DOI:** 10.31744/einstein_journal/2026AO1837

**Published:** 2026-05-27

**Authors:** Ana Claudia Pelissari Kravchychyn, Raquel Munhoz da Silveira Campos Mônico, Bárbara Dal’Molin, Yasmin Alaby Martins Ferreira, Anna Luiza Maia Balbino, Helton de Sá Souza, Lila Missae Oyama, Lian Tock, Ana Raimunda Dâmaso

**Affiliations:** 1 Universidade Federal de Viçosa Laboratory of Clinical Analysis and Genomics Department of Nutrition and Health Viçosa MG Brazil Department of Nutrition and Health, Laboratory of Clinical Analysis and Genomics, Universidade Federal de Viçosa, Viçosa, MG, Brazil.; 2 Universidade Federal de São Paulo Department of Biosciences Santos SP Brazil Department of Biosciences, Universidade Federal de São Paulo, Santos, SP, Brazil.; 3 Universidade Federal de São Paulo Santos SP Brazil Post Graduate Program of Interdisciplinary Health Sciences, Universidade Federal de São Paulo, Santos, SP, Brazil.; 4 Universidade Federal do Paraná Curitiba PR Brazil Universidade Federal do Paraná, Curitiba, PR, Brazil.; 5 Universidade Federal de São Paulo São Paulo SP Brazil Post Graduate Program of Nutrition, Universidade Federal de São Paulo, São Paulo, SP, Brazil.; 6 Universidade Federal de Viçosa Department of Physical Education Viçosa MG Brazil Department of Physical Education, Universidade Federal de Viçosa, Viçosa, MG, Brazil.

**Keywords:** Sweet sugar beverage, Ultraprocessed food, Obesity, Adolescents, Leptin/adiponectin ratio

## Abstract

**Objective:**

To evaluate the relationship between dietary patterns and inflammatory markers in Brazilian adolescents with obesity.

**Methods:**

A cross-sectional analysis was conducted among 70 post-pubertal adolescents aged 15–19 years of both sexes who were classified as obese according to the World Health Organization criteria. Blood samples were collected to determine leptin and adiponectin levels using enzyme-linked immunosorbent assay kits, and the leptin/adiponectin ratio was calculated. Anthropometric measurements and body composition were assessed. Food intake was evaluated using a 24-hour dietary recall and categorized according to the NOVA classification into: fresh or minimally processed foods, processed culinary ingredients, processed foods, and ultra-processed foods. Participants were grouped according to sugar-sweetened beverage intake into intakers (n=41) and non-intakers (n=29).

**Results:**

Adolescents who consumed sugar-sweetened beverages had higher fat-free mass, body weight, resting metabolic rate, and pro-inflammatory leptin/adiponectin ratio. They also showed greater intake of total grams, energy, proteins, lipids, cholesterol, carbohydrates, fiber, and saturated and polyunsaturated fats from ultra-processed foods. Additional analyses revealed that ultra-processed food intake was associated with increased body weight and higher waist and neck circumferences, which are relevant predictors of pro-inflammatory status.

**Conclusion:**

The findings suggest that sugar-sweetened beverage consumption and high ultra-processed food intake are associated with unfavorable anthropometric indicators and increased pro-inflammatory biomarkers, reflected by higher leptin/adiponectin ratios in adolescents with obesity.

**ClinicalTrials.gov Identifier:**

RBR-6txv3v.

## In Brief

Adolescents with obesity showed a higher leptin/adiponectin ratio associated with the consumption of sugar-sweetened beverages and ultra-processed foods. These findings indicate a more unfavorable inflammatory and metabolic profile related to diet quality.

## Highlights

■ Sugar-sweetened beverages are linked to a poorer inflammatory profile in adolescents.
■ Ultra-processed food intake is associated with a higher leptin/adiponectin ratio.
■ The leptin/adiponectin ratio reflects metabolic risk in adolescent obesity.
■ Dietary quality influences inflammatory hormonal markers.

## Graphical abstract

**Figure:**
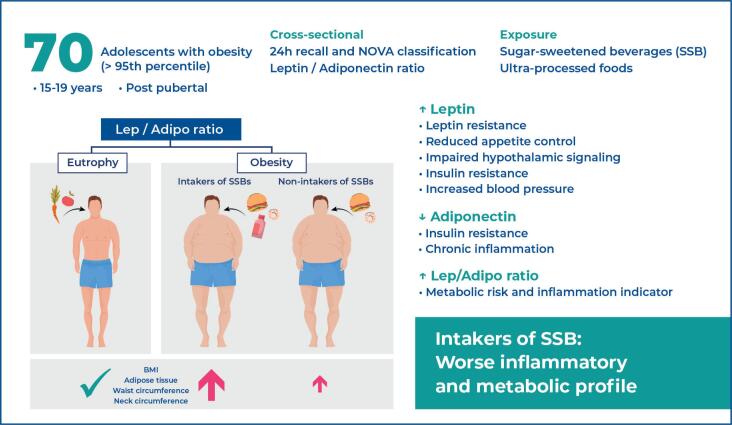

## INTRODUCTION

The alarming prevalence of obesity in children and adolescents reflects the nutritional transition occurring in the population, which involves both dietary patterns and the family environment. Dietary patterns among the pediatric population have changed over time, with increased consumption of energy-dense foods that are low in fiber and micronutrients but rich in fat and refined sugar.^(1,2)^ These foods are generally highly processed and were developed to produce ready-to-eat, durable, convenient, and profitable products. Recent studies increasingly highlight the association between greater consumption of highly processed foods and the development of obesity.^(1–3)^

An important category of ultra-processed foods (UPFs) is sugar-sweetened beverages (SSBs), which may contribute substantially to the epidemic of overweight and obesity in children and adolescents because of their high added sugar content, low satiety, and poor nutritional contribution to total energy intake.^(4,5)^ As body fat increases, the inflammatory profile and hormonal regulation of hunger and metabolism change. In this context, the expression and secretion of leptin and adiponectin change and are modulated by body mass index, adipocyte size, distribution, and function, as well as by sex, dietary components, and inflammatory cytokines.^(6,7)^

Leptin plays an important role in regulating energy balance, food intake, and body weight. Patients with obesity often develop leptin resistance, which impairs the neuroendocrine regulation of energy homeostasis.^(7–9)^ Furthermore, hyperleptinemia has been associated with pro-inflammatory conditions in adolescents with obesity. Adiponectin, in contrast, is secreted by adipocytes and plays a beneficial role in glucose and lipid metabolism by reducing the inflammatory state. This adipocytokine suppresses hepatic glucose production and improves insulin sensitivity.^(10)^ However, adiponectin levels decrease in adolescents with obesity, occurring in parallel with hyperleptinemia. These findings suggest that both hormones act as key mediators of obesity-related inflammatory processes in this population. In fact, the leptin/adiponectin ratio is directly associated with glucose metabolism and adipose tissue function and has been proposed as a potential biomarker of inflammation.^(11, 12)^

Studying the relationship between the pathophysiological aspects of these conditions and dietary intake remains important, because dietary patterns can trigger and amplify inflammation-related factors that worsen obesity and contribute to the development of comorbidities. Few studies have examined the relationship between these hormones and the consumption of SSBs, particularly in pediatric populations. In addition, limited evidence has evaluated the combined impact of SSB and UPF consumption on inflammatory biomarkers in Brazilian adolescents. We hypothesized that a higher intake of SSBs and UPFs would be associated with elevated inflammatory markers, including an increased leptin/adiponectin ratio.

## OBJECTIVE

This study evaluate the impact of sugar-sweetened beverages and ultra-processed foods intake on the leptin/adiponectin ratio, an important biomarker of inflammation, in adolescents with obesity.

## METHODS

This cross-sectional observational study included post-pubertal adolescents recruited from clinical and community settings, including outpatient clinics and local schools, to ensure a representative sample and improve reproducibility. Seventy post-pubertal adolescents with obesity aged 15-19 years of both sexes participated in the study. The inclusion criteria were Tanner stage V,^(13)^ primary obesity, and body mass index greater than the 95th percentile according to the World Health Organization reference growth charts. The exclusion criteria included use of oral contraceptives, corticosteroids, or anti-epileptic drugs, history of renal disease, alcohol consumption, smoking, and secondary obesity resulting from endocrine disorders. The study followed the principles of the Declaration of Helsinki and received approval from the research ethics committee of the *Universidade Federal de São Paulo* (CAAE: 45564721.4.0000.5505; # 4.846.311). All procedures were explained to the guardians of the participants, and written informed consent was obtained.

Participants underwent an initial assessment to collect sociodemographic and health information provided by the adolescents or their guardians. This assessment included questions on birth history, weight, duration of breastfeeding, race, history of childhood respiratory diseases, rhinitis, or sinusitis, family history of smoking, asthma and obesity, socioeconomic status, and pubertal stage. A complete clinical evaluation, blood tests, and blood pressure measurements were performed by an endocrinologist. On the scheduled assessment day, blood samples were collected, and anthropometric and body composition measurements were obtained. A 24-hour dietary recall was also administered to assess food intake.

### Serum analysis

Blood samples were collected after an overnight fast at approximately 08:00 h in the outpatient clinic. After collection, samples were centrifuged for 10 min at 5,000 rpm and stored at −80°C for subsequent analyses. Blood collection was performed by a trained and qualified technician. Leptin and adiponectin concentrations were measured using commercial immunoassay kits from eBioscience (San Diego, CA, USA) and R&D Systems (Minneapolis, MN, USA), according to the instructions of the manufacturers. The pro-inflammatory leptin/adiponectin ratio was then calculated.

### Energy intake and NOVA classification

Food intake was assessed using a 24-hour dietary recall. The data were analyzed using the Brazilian Food Composition Table,^(14)^ Food Composition Tables,^(15)^ and the Chemical Food Composition Table,^(16)^ in addition to the nutritional information obtained from the official websites of the products reported by the participants.

Food items were categorized according to the NOVA classification system:^(17)^ unprocessed or minimally processed foods; processed culinary ingredients; processed foods; and UPFs. This classification provides a practical framework to evaluate the relationship between food processing, dietary quality, and its potential impact on metabolic and inflammatory biomarkers. After determining the level of food processing, adolescents were classified according to SSB intake into two groups: intakers (n=41) and non-intakers (n=29).

### Anthropometric measurements and body composition

Body weight was measured with participants wearing light clothing and no shoe using a Filizola^®^ scale, with a precision of 0.1kg and a maximum capacity of 180kg. Height was measured using a wall-mounted height board (Sanny^®^) to the nearest 0.1cm. Body mass index was calculated as body weight (kg) divided by height (m)^2^. Waist circumference and neck circumference were measured using a flexible, non-elastic measuring tape. Body composition was assessed using bioelectrical impedance analysis. Basal metabolic rate was estimated based on body composition using the BIODYNAMICS 310e device (TBW^®^), which shows a correlation of R=0.98 with hydrostatic weighing and a resistance reading accuracy of ±1%.

### Statistical analysis

Statistical analyses were performed using SPSS version 21.0 (SPSS Inc., Chicago, IL, USA). A generalized linear model with an appropriate distribution (gamma or linear), determined using the Akaike information criterion, was used to evaluate differences between groups according to SSB intake. Sidak post hoc tests were applied for pairwise comparisons. The generalized linear model was also used to examine associations among variables, with body mass, neck circumference, and waist circumference included as dependent variables. Intake from each NOVA category was included as an independent variable. Statistical significance was defined as p≤0.05. To account for potential confounders, the model was adjusted for sex, socioeconomic status, and physical activity. When adjustment was not possible, this limitation was acknowledged and justified.

Statistical power was calculated using G*Power version 3.1. The analysis assumed an alpha level of 0.05, an effect size of 0.41, leptin/adiponectin ratio values from our database, two groups and two time points, and a total sample size of 70 participants. The resulting statistical power was 0.96.

## RESULTS

Table 1 presents the analysis of body composition, circumferences, leptin, adiponectin, and leptin/adiponectin ratio values. Adolescents who consumed SSBs exhibited significantly higher fat-free mass (kg), body weight, resting metabolic rate, and pro-inflammatory leptin/adiponectin ratio than non-intakers, highlighting the potential metabolic and inflammatory impact of SSB consumption.

**Table 1:** Analysis of anthropometric variables, body composition, adiponectin, and leptin in intakers and non-intakers of sugar-sweetened beverages

	Non-intakers (n=29)	Intakers (n=41)	p value
WC (cm)	107.59±7.49	111.00±10.90	0.136
BF (%)	37.34±4.79	38.02±5.49	0.543
FFM (kg)	66.31±7.17	70.43±12.79*	0.036
FFM (%)	62.73±4.93	62.01±5.20	0.501
Body weight (kg)	105.72±8.66	114.35±18.63*	0.016
NC (cm)	39.19±2.37	40.12±4.34	0.171
RMR (kcal)	2009.40±218.73	2141.28±388.60*	0.020
Leptin (ng/mL)	42.35±23.67	49.23±22.19	0.230
Adiponectin (μg/mL)	2.58±1.51	2.60±2.10	0.966
Leptin/adiponectin ratio	21.6±21.8	35.9±39.6*	0.034

Data are presented as mean±standard deviation.

* Significant difference compared with non-intakers. P-values compare intakers versus non-intakers.

WC: waist circumference; BF: body fat; FFM: fat-free mass; NC: neck circumference; RMR: rest metabolic rate.

Table 2 presents food intake according to the level of food processing for SSB intakers and non-intakers. Adolescents who consumed SSBs had significantly higher intake of grams, energy (kcal), proteins, lipids, cholesterol, carbohydrates, fiber, and both saturated and polyunsaturated fats from ultra-processed foods. They also showed higher cholesterol intake from processed foods than non-intakers. These differences were statistically significant and clinically relevant, particularly for energy intake and the leptin/adiponectin ratio.

**Table 2:** Food intake by NOVA classification according to sugar-sweetened beverage consumption groups

		Non-intakers (n=29)	Intakers (n=41)	p value
In Natura	Total grams	828.25±418.54	756.88±383.56	0.489
	Energy intake (kcal)	822.53±379.09	908.75±587.02	0.503
	Protein (g)	55.62± 29.81	67.08±56.53	0.324
	Lipids (g)	19.53±16.94	28.45±29.41	0.143
	Cholesterol (g)	227.41±203.78	267.22±184.15	0.383
	Carbohydrates (g)	101.61±63.58	98.33±67.38	0.846
	Fiber (g)	16.78±14.35	17.52±16.59	0.843
	SFAs (g)	6.63±5.51	11.44±13.05	0.051*
	MUFAs (g)	6.69±5.79	12.99±12.97	0.014*
	PUFAs (g)	2.15±2.92	3.47±14.35	0.092
Processed culinary ingredient	Total grams	30.84±20.25	29.41±22.17	0.801
	Energy intake (kcal)	199.80±105.88	201.90±141.04	0.948
	Protein (g)	0.09±0.05	0.07±0.05	0.640
	Lipids (g)	18.32±10.37	20.41±15.12	0.527
	Cholesterol (g)	11.58±2.68	27.02±14.24	0.414
	Carbohydrates (g)	20.72±15.76	12.90±11.07	0.523
	Fiber (g)	0.00±0.00	0.00±0.00	—
	SFAs (g)	2.90±1.63	4.07±4.29	0.072
	MUFAs (g)	7.09±6.28	6.10±5.24	0.504
	PUFAs (g)	8.19±4.31	10.23±7.02	0.144
Processed	Total grams	198.13±219.24	337.43±392.76	0.093
	Energy intake (kcal)	342.20±307.28	382.10±368.22	0.643
	Protein (g)	14.15±15.12	15.36±17.60	0.744
	Lipids (g)	11.32±14.71	14.98±20.91	0.385
	Cholesterol (g)	37.98±26.86	106.51±97.65	0.013*
	Carbohydrates (g)	39.97±40.61	52.78±50.36	0.268
	Fiber (g)	2.01±2.04	2.30±1.95	0.546
	SFAs (g)	5.92±5.36	4.87±9.71	0.677
	MUFAs (g)	4.68±4.45	3.23±4.60	0.400
	PUFAs (g)	1.71±1.79	2.51±4.33	0.297
Ultra-processed	Total grams	180.00±192.73	658.06±373.09	<0.000*
	Energy intake (kcal)	619.29±554.55	1349.42±982.50	<0.000*
	Protein (g)	18.88±18.90	30.95±20.68	0.013*
	Lipids (g)	22.28±26.98	39.37±25.50	0.007*
	Cholesterol (g)	31.42±22.57	55.36±43.64	0.024*
	Carbohydrates (g)	55.81±61.96	175.49±164.17	<0.000*
	Fiber (g)	3.22±4.93	6.23±4.84	0.004*

Data are presented as mean±standard deviation. P-values compare intakers *versus* non-intakers.

* Significant difference between groups.

SFA: saturated fatty acid; MUFA: monounsaturated fatty acid; PUFA: polyunsaturated fatty acid.

Finally, positive and statistically significant associations were observed between UPF intake and body weight, waist circumference, and neck circumference, highlighting the role of UPFs in influencing anthropometric and inflammatory markers in adolescents with obesity (Table 3).

**Table 3:** Association between ultra-processed food intake and anthropometric measures

		β value	Exp(B)	95% CI	p value
Body weight (kg)					
Unprocessed (g)		0.00002	1.000	1	0.626
Processed culinary ingredients (g)		–0.001	0.999	0.997–1	0.092
Processed (g)		–0.000105	1.000	1	0.021
Ultra-processed (g)		0.000115	1.000	1	0.004*
Waist circumference (cm)					
	Unprocessed (g)	0.002	1.002	0.996–1.008	0.543
	Processed culinary ingredients (g)	–0.033	0.968	0.865–1.083	0.569
	Processed (g)	–0.005	0.995	0.988–1.002	0.146
	Ultra-processed (g)	0.007	1.007	1.001–1.013	0.018*
Neck circumference (cm)					
	Unprocessed (g)	0.000015	1.000	0.998–1.002	0.990
	Processed culinary ingredients (g)	–0.011	0.989	0.947–1.033	0.629
	Processed (g)	–0.002	0.998	0.996–1.001	0.220
	Ultra-processed (g)	0.003	1.003	1–1.005	0.022*

* Statistically significant associations for variables influencing body weight, waist circumference, and neck circumference.

95%CI: 95% confidence interval.

## DISCUSSION

**Figure 1:**
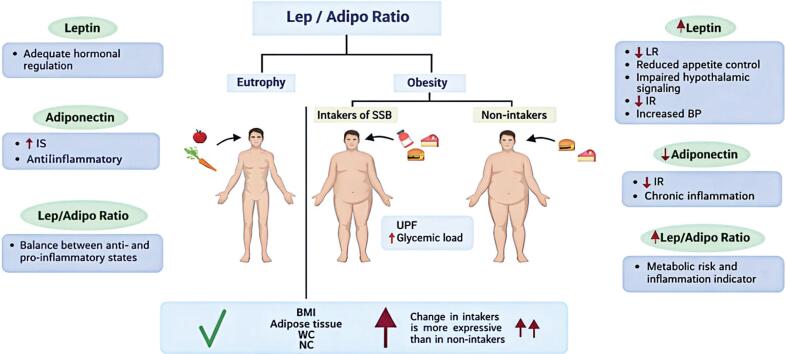
Relationship between leptin/adiponectin ratio, nutritional status, and sugar-sweetened beverage intake among adolescents

Source: Created using the BioRender tool. Available from: https://www.biorender.com/

This study focused on food quality and tested the hypothesis that UPF intake and SSB consumption are associated with alterations in variables involved in the pathophysiology of obesity in adolescents. The results showed that higher consumption of SSBs and UPFs was associated with an increased pro-inflammatory biomarker, the leptin/adiponectin ratio, as well as with higher body mass and greater waist and neck circumferences. These findings support the role of diet in modulating inflammatory status in adolescents with obesity (Figure 1).

Lep/Adipo: leptin/adiponectin; SSB: sugar-sweetened beverage; UPF: ultra-processed food; BMI: body mass index; WC: waist circumference; NC: neck circumference; IS: insulin sensitivity; IR: insulin resistance; BP: blood pressure; LR: leptin resistance.

Routine intake of UPFs and saturated fats plays a key role in the development of obesity by promoting hypothalamic inflammation and reducing hypothalamic responsiveness to satiety signals.^(11,18,19)^ Recent evidence suggests that UPF consumption can directly disrupt neural circuits involved in appetite regulation, independently of adiposity, thereby contributing to overconsumption and weight gain.^(20)^ To our knowledge, this study represents the first investigation in Brazil, and among the first in Latin America, to evaluate the relationship between high UPF and SSB intake and inflammatory biomarkers such as the leptin/adiponectin ratio in adolescents with obesity.

Food intake and obesity are influenced not only by caloric content but also by the degree of food processing.^(21–23)^ In addition to their low nutritional value, UPFs may stimulate appetite because of their high glycemic load, whereas minimally processed foods promote greater satiety.^(24–26)^ Sugar-sweetened beverages as liquid UPFs, generate weaker satiety signals than solid foods.^(27)^

Adipokines play a central role in the pathophysiology of obesity because of variations associated with nutritional status.^(12,28)^ Leptin stimulates anorexigenic pathways and regulates inflammatory processes. However, in obesity, leptin resistance develops, leading to hyperleptinemia and promoting a pro-inflammatory state, insulin resistance, and elevated blood pressure.^(12,29,30)^ In contrast, adiponectin, which improves insulin sensitivity and metabolic regulation, decreases in adolescents with obesity. This imbalance results in a state of hyperleptinemia with concomitant hypoadiponectinemia and reduced satiety signaling.

The leptin/adiponectin ratio reflects this pathophysiological context and provides a more integrated marker than isolated leptin or adiponectin measurements, showing associations with body mass index, weight, glycemic markers, insulin resistance, and other cardiometabolic comorbidities.^(12,28,30–32)^

In the present sample, UPF intake represented 39% of total energy intake among non-consumers of SSBs and 69% among SSB consumers, indicating a 30% higher intake in adolescents who consumed SSBs. This difference may help explain the observed metabolic differences between groups. These findings align with other Brazilian studies reporting associations between UPF consumption and overweight or abdominal obesity.^(3,33,34)^

Sugar-sweetened beverages remain the primary source of added sugars in adolescent diets. Fructose from SSBs promotes hepatic lipogenesis, fat accumulation, insulin resistance, and the release of pro-inflammatory cytokines, thereby increasing the risk of type 2 diabetes and cardiovascular disease.^(35)^ The present findings reinforce the need for public health strategies to reduce UPF and SSB consumption among adolescents, including school-based nutritional interventions and broader policy initiatives aimed at promoting healthier dietary patterns and preventing obesity and related metabolic disorders.

Although the sample consisted exclusively of adolescents with obesity, differences in SSB consumption were associated with higher UPF intake, greater total energy intake, and higher cholesterol consumption. The fiber-to-carbohydrate ratio was lower among SSB consumers, reflecting a less balanced dietary pattern that may exacerbate obesity and increase the risk of chronic disease progression.

This study has some limitations, including the use of a single 24-hour dietary recall, which may not reflect habitual intake. However, strengths include the integration of dietary and biochemical markers and the high statistical power achieved.

## CONCLUSION

In summary, the present investigation suggests a relationship between sugar-sweetened beverages intake and the high consumption of ultra-processed foods among adolescents with obesity, which may contribute to differences in pro-inflammatory biomarkers, reflected by a higher leptin/adiponectin ratio in this population. All participants had a diagnosis of obesity, and these findings highlight the importance of diet quality in preventing pro-inflammatory states and comorbidities commonly associated with obesity in adolescents. These results support the development of guidelines and preventive interventions aimed at reducing sugar-sweetened beverages and ultra-processed foods consumption among adolescents.

## Data Availability

The underlying data are contained within the manuscript.
